# MediScan: A Framework of U-Health and Prognostic AI Assessment on Medical Imaging

**DOI:** 10.3390/jimaging10120322

**Published:** 2024-12-13

**Authors:** Sibtain Syed, Rehan Ahmed, Arshad Iqbal, Naveed Ahmad, Mohammed Ali Alshara

**Affiliations:** 1 School of Computing Sciences, Pak-Austria Fachhochschule Institute of Applied Sciences and Technology (PAF-IAST), Mang, Haripur 22621, Khyber Pakhtunkhwa, Pakistan; sibtainshah621@gmail.com (S.S.); rehanahmedahmed007@gmail.com (R.A.); 2 Sino-Pak Center for Artificial Intelligence (SPCAI), Pak-Austria Fachhochschule Institute of Applied Sciences and Technology, Mang, Haripur 22621, Khyber Pakhtunkhwa, Pakistan; 3 College of Computer and Information Sciences, Prince Sultan University, Riyadh 11586, Saudi Arabia; nahmed@psu.edu.sa (N.A.); malshara@psu.edu.sa (M.A.A.)

**Keywords:** convolutional neural networks, disease recognition, healthcare application, image processing, Large Language Model (LLM), malignancy classification

## Abstract

With technological advancements, remarkable progress has been made with the convergence of health sciences and Artificial Intelligence (AI). Modern health systems are proposed to ease patient diagnostics. However, the challenge is to provide AI-based precautions to patients and doctors for more accurate risk assessment. The proposed healthcare system aims to integrate patients, doctors, laboratories, pharmacies, and administrative personnel use cases and their primary functions onto a single platform. The proposed framework can also process microscopic images, CT scans, X-rays, and MRI to classify malignancy and give doctors a set of AI precautions for patient risk assessment. The proposed framework incorporates various DCNN models for identifying different forms of tumors and fractures in the human body i.e., brain, bones, lungs, kidneys, and skin, and generating precautions with the help of the Fined-Tuned Large Language Model (LLM) i.e., Generative Pretrained Transformer 4 (GPT-4). With enough training data, DCNN can learn highly representative, data-driven, hierarchical image features. The GPT-4 model is selected for generating precautions due to its explanation, reasoning, memory, and accuracy on prior medical assessments and research studies. Classification models are evaluated by classification report (i.e., Recall, Precision, F1 Score, Support, Accuracy, and Macro and Weighted Average) and confusion matrix and have shown robust performance compared to the conventional schemes.

## 1. Introduction

Healthcare has evolved from a quaint industry to a billion-dollar market over the last few decades. During the past years, data-driven decisions have been implemented in multiple domains such as Healthcare, Character Recognition, and water resources [1,2,3]. With the rapid advancement in IT in healthcare, the increasing demands for quality assurance, financing, collection, and storage of medical data are becoming more complex. During the past 50 years, advanced tools and strategies have been followed for collecting and storing medical data to keep pace with the changes and growth of the modern healthcare system. These significant advancements have led to modern computer technology being an integral part of the healthcare system.

The healthcare system is undergoing a continuous transition from E-health to U-health, characterized by the emergence of smart healthcare and other associated research works linked with technological advancements [4,5,6]. E-health is referred to as a medical service that uses Information Technology (IT) to connect computer servers and medical institutions to improve the delivery of medical information and health [7]. On the other side, a U-health platform uses IT technology to improve medical systems for providing medical and health-related information, knowledge, products, and services to consumers such as medical institutions, individuals, and companies [8]. Additionally, it allows consumers to check their medical records and health status anytime, anywhere. Smart healthcare refers to the integration of healthcare-related services with IT to provide health-related information, systems, equipment, and platforms that concern personal health and healthcare [9].

Although the doctors are medical personnel, there is still a risk and margin of human error in writing a personalized health prescription or diagnosing a disease for their respective patients. To apprehend this margin of error, a framework is proposed that allows the doctor to investigate a medical report of the patient from the laboratory with an AI assessment module. The proposed framework aims to efficiently classify the disease by examining the medical images and giving personalized precautions for the specific disease. This allows the doctor to better analyze the patient’s health, which minimizes the margin of human error.

The respective framework proposes a user-friendly U-health platform that allows patients and doctors to track patient’s medical history, intercommunicate with each other, and book a doctor’s appointment. The proposed framework allows the patient to analyze different doctors’ profiles, connect with a pharmacy, and make payments online, thus removing the communication bridge between patients and doctors, and improving the medical healthcare systems. The research aims to propose a solution to improve healthcare management by integrating hospitals, laboratories, and pharmacies onto a single platform, providing comprehensive healthcare solutions through state-of-the-art AI-empowered medical settings. The proposed framework utilizes AI for patient risk assessments by identifying multiple malignancies and fractures in the human body. The AI module utilizes CT scan images to identify lung cancer and kidney cancer, uses MR images for identifying brain tumors, X-ray images for fracture types, and microscopic images for recognizing skin cancer. The proposed U-health platform also facilitates doctors for more accurate diagnoses and timely treatments of the patient. The respective framework allows the patient and doctor to track medical history, minimizes the communication gap, and provides health-related information, knowledge, and other services to consumers.

The proposed study is organized into different sections. Section 2 presents similar frameworks and techniques proposed. Section 3 explains the MediScan architecture, while Section 4 covers the AI modules used in the study. Section 5 explains model evaluators, while Section 6 discusses the results and compares them with existing models. Section 7 shows the concluding remarks and future working of the proposed framework.

## 2. Literature Review

Healthcare systems are experiencing increasing strains due to population growth and, hence, a rise in patient numbers. Consequently, it is essential to establish intelligent healthcare systems by integrating IT innovations into the healthcare infrastructure. Medical image processing techniques are crucial for an early-stage diagnosis and disease monitoring during treatment. The proposed framework has integrated different medical imaging AI models for identifying diseases from different imaging modalities such as CT scans, X-rays, microscopic images, and MRIs.

Timely lung cancer diagnosis and treatment are vital for increasing the survival rate of 5 years owing to the heterogeneity and invasiveness of the disease [10]. Recently, a systematic meta-analysis and review was conducted for achieving diagnostic accuracy from DL approaches for lung cancer diagnosis, and results have shown that the pooled sensitivity and specificity coasted by these approaches are 93% and 68%, respectively [11]. Research has also shown that deep learning models can also be used in diagnosing fractures. In 2018, a study proposed to utilize a deep CNN model for detecting fractures from plain wrist radiographs [12]. Multiple studies have also been employed for the classification of brain tumors. It is crucial to analyze MRI-based brain images before a surgical procedure, and extrapolating information can help save time and reduce pain. In 2020, a study was conducted for Multi-modal Brain Tumor Classification using VGG-16 and VGG-19 on different BraTS datasets [13]. In humans, skin cancer is considered to be one of the most deadly diseases. Melanoma and nevus lesions share high similarity due to which physicians usually spend a lot of time examining their samples. A data-driven approach could help physicians classify these lesions more efficiently.

In 2023, SkinLesNet was presented, which is a multi-layer deep CNN model used to classify three types of skin lesions, including melanoma, using a dataset of smartphone images [14]. Kidney tumor is also considered to be one of the most prevalent diseases in our society. It is among the seven most common types of tumor found in men and women, globally. Early detection of kidney cancer can be significant in reducing death rates and overcoming the tumor. As opposed to traditional diagnosis, AI can save diagnosis time, reduce cost, help in improving tests’ accuracy, and reduce the doctor’s workload. In 2022, a study was proposed to classify kidney tumors in a human through 2D CT scan images by employing a 2D CNN-6 model, which performed robustly and achieved 97% accuracy [15].

Table 1 presents a comprehensive overview of the past AI-based techniques used to detect malignancy in different parts of humans through medical images. The studies aim to improve the healthcare industry. Although the past research results have suggested AI potential in medical imaging, there are still many research challenges to be addressed [11,12,13,14].

## 3. Proposed AI-Enabled U-Health Framework

### 3.1. System Modeling

**Figure 1:**
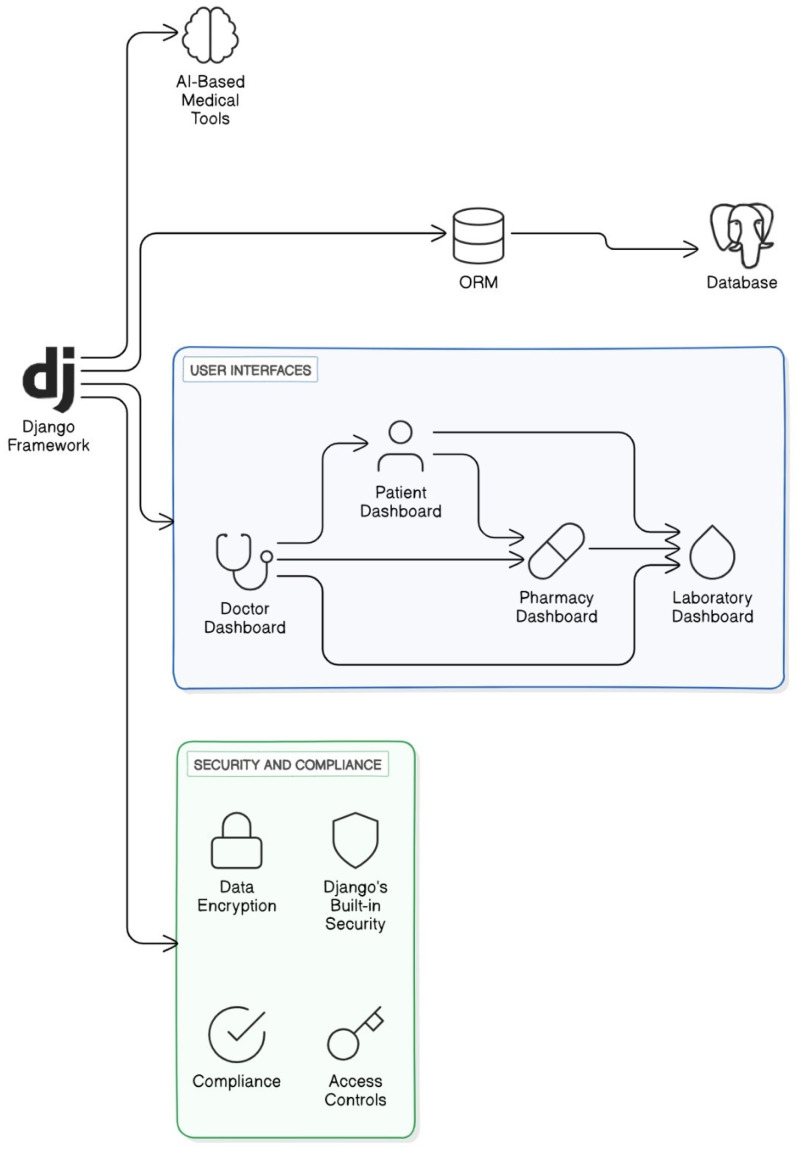
Graphical scheme of the system architecture.

Due to its robust, scalable, and secure architecture, the Django framework is very beneficial for the healthcare system’s development. Django offers many features that are critical and play a pivotal role in securing and managing healthcare data. In the Django database, data retrieval works through Object-Relational Mapping (ORM). Therefore, ORM works for retrieving patient records, laboratory results, and pharmaceutical inventories. Due to the modular design of Django, it works with the integration of different components like AI-based medical tools, laboratory dashboards, doctor dashboards, and patient and pharmacy dashboards as illustrated in Figure 1.

In our system, a doctor can register themselves by creating a profile. Then they will wait for the confirmation of their account verification from the hospital. After the hospital’s approval that they belong to that hospital, the doctor will be allowed to start their work. When a patient makes an appointment with the doctor and discusses his situation, the doctor will prescribe a test to the patient, which will be automatically sent to the laboratory and patient. The laboratory dashboard has the test list associated with the doctor and patient. Because after the test submission, both can view the test reports.

The Django application primarily focuses on security due to its reliability especially in the healthcare industry. It is important to secure sensitive data of patients, which is achieved by built-in security features for SQL injection prevention, cross-site scripting prevention, and cross-site request forgery protection. Due to Django’s compliance with GDPR and HIPAA standards, it guarantees that the system follows strict regulatory criteria that are required for holding the patient’s confidence. Django’s extensive administration interface provides strong management tools at the backend and to healthcare administration, who can modify the admin interface according to their requirements, enabling efficient user control and reporting features. The sub-technologies covered by the proposed system modeling are as shown in Table 2.

**Figure 2:**
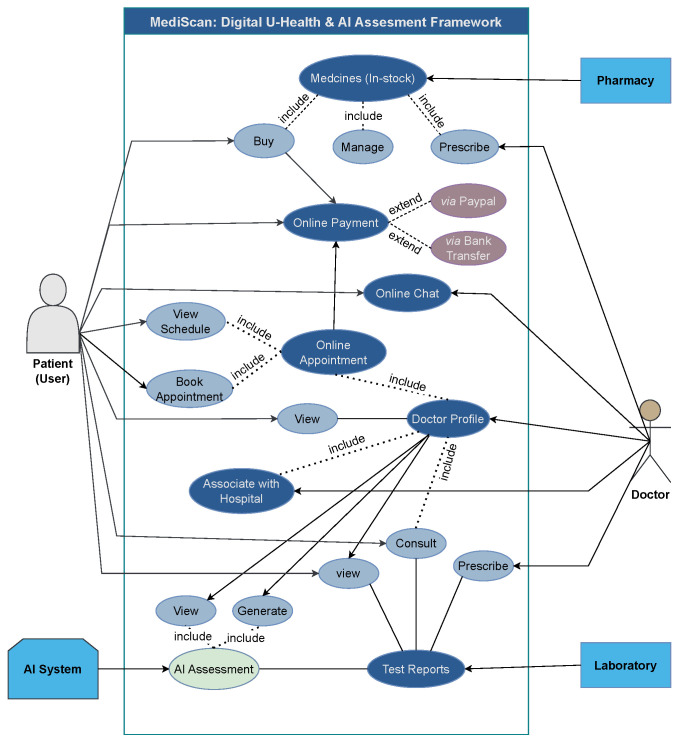
Graphical scheme of use cases in the proposed framework.

The use case of the proposed framework is illustrated in Figure 2, providing an overview of the relation between actors and features of the proposed U-health platform.

#### 3.1.1. Patient Functionality

Patients play a central role in the hospital management system. The patient can book appointments online through the system by checking the doctor’s profile, which also increases the confidence of the patient. To check the availability of the doctor, patients can check the doctor’s calendar, which includes other appointments. Patients can also read the doctor’s profile to check their educational and experience records as well.

Patients can keep track of their health state without visiting the hospital because they can access their test reports through the system. The system also has chat accessibility between doctors and patients, where patients can discuss their health concerns with doctors through the online consultation supported by the system. To improve the accessibility of healthcare services and the overall experience, the integration of these features promotes the patient-centered approach.

#### 3.1.2. Doctor Functionality

Doctors play an extremely important role in this system. Each profile created by the doctors is connected to the hospital that they are associated with. That will increase the authenticity of doctors. Once the doctor receives the reports from lab directly in their system, then they can analyze reports with the help of the AI assessment tool, which is able to identify the malignancy of the patient through the medical image and provide appropriate risk assessment and insights to the doctor. The doctor can than prescribe the right medication to the patient, improving the overall accuracy and efficiency of diagnostic processes.

In Figure 2, the doctor is able to view the patient test report received from the laboratory and write a prescription for the patient. On the other hand, the patient can also have the option to consult the doctor based on received test reports from the laboratory. Along with that, the doctor is also able to generate and view AI-generated test reports, which include the AI-identified disease and AI assessment for the patient.

#### 3.1.3. Laboratory

The laboratory is important to the system’s information flow. Medical tests are performed in the laboratories, and test results must be uploaded on the system. Test reports are received by the patient and doctors simultaneously after uploading to the system. This instant accessibility to the reports can allow doctors to move forward with the diagnosis and treatment plans.

#### 3.1.4. Pharmacy

It is the pharmacy’s responsibility to keep an eye on the system’s medicine inventory and its sales. When doctors prescribe medicine to a patient, it will automatically be forwarded to the pharmacy. Patients will buy medicine online without having physical interaction with the pharmacy. The pharmacy can also streamline its operations and guarantee that the patient can obtain the prescribed medication by using the online system, as well as monitor its supply and sales.

#### 3.1.5. AI Assessment Module

The AI report assessment toll available on doctor’s dashboard is one of this system’s most important features. By utilizing deep learning algorithms, this tool analyzes the patient’s test reports to give doctors more accurate diagnostic information. The AI system can detect the disease and also provide precautions and recommendations to the doctors that are helpful in diagnosis and prescription to the patient. This AI can help the doctors to make quicker decisions and more accurate medical judgments.

## 4. Proposed AI Module Architecture

### 4.1. Bone Fracture Classification

The data curation was performed by scrapping Google images for the respective types of bone fractures. The accumulated dataset consists of 1129 X-ray images. The number of classes were 10 and for each class of fracture, more than 150 images were composed to make the data more balanced. The images were first transformed to float32 (floating point representation) to apply standard scaling on the images by dividing each pixel value by 255.0 to ensure numerical stability and uniformity in the data. Thus, the resultant pixels’ values of images were in between the range of [0, 1]. The images were also resized to 256×256 as it ensures a uniform input size for AI models, improves computational efficiency, and standardizes data for consistent processing. The data were then split by 90:10 ratio into the training and testing datasets comprised of 1017 and 112 images, respectively.

For training, the CNN model was implied on the given dataset. The layers and neurons along with other convolution hyper-parameters were selected through the trial-and-error technique; this process ensures the optimization of the model on the given dataset and accumulation of the best results. The hyper-parameters for the proposed model are represented in the following Table 3.

**Figure 3:**
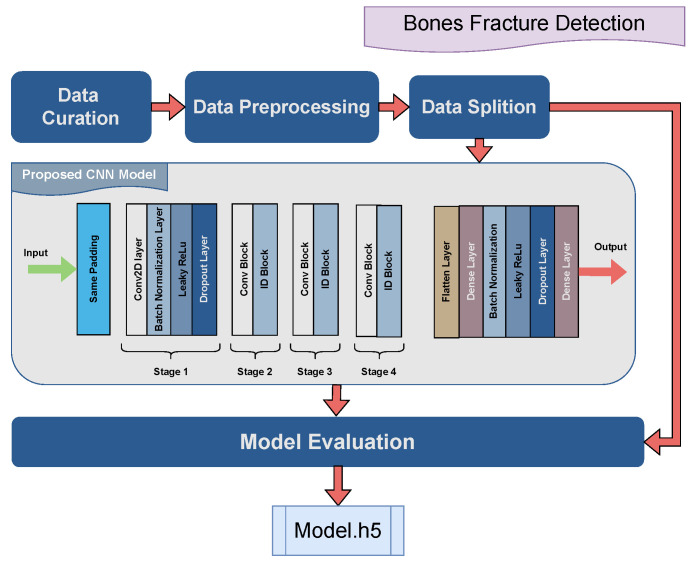
Graphical illustration of proposed AI bones fracture detection model.

Figure 3 illustrates the layers’ architecture of the proposed CNN model. The input layer shape is 256×256 with three color channels (RGB). The architecture consists of four convolutional layers, batch normalization, leaky ReLU activation, and dropout regularization. Leaky ReLU, a variation of the ReLU function, allows minimal non-zero gradient whenever the input is negative and is mathematically expressed as:

(1) $$g\left(x\right)=\left\{\begin{array}{cc} x & \mathrm{if}\;x>0\\ \alpha x & \mathrm{otherwise}. \end{array}\right.$$

The convolutional layers have 32, 64, 128, and 256 numbers of filters, respectively. The kernel size is 3 × 3, while a stride of 2 is used for downsampling and padding is applied to maintain spatial dimensions. The mathematical expression for the output size of a convolutional layer is:

(2) $$\mathrm{Output}=\frac{i-f+2p}{s}+1$$

where *i*, *f*, *p*, and *s* represent the input size, filter size, padding size, and stride size.

Then a flatten layer is implied to convert the convolutional output into a vector to be fed into the dense layers. A single dense layer with 100 units is implied, followed by batch normalization, leaky ReLU activation, and dropout. This layer helps in learning higher-level features from the flattened representation. For the output, a dense layer with 10 units (or neurons) was imposed with softmax as an activation function to produce probabilities for each class:

(3) $$\mathrm{softmax}{\left(z\right)}_{i}=\frac{e^{z_{i}}}{\sum_{j=1}^{K}e^{z_{j}}}.$$

where $z_{i}$ is the ith value of input vector *z*, and *K* is the number of elements in the vector.

### 4.2. Lung Nodule Detection

The dataset is composed by collecting multiple lung cancer images for respective types of cancer. The accumulated dataset consists of 995 CT scan images of different patients. The images are taken in jpg or png format rather than dcm format. The data consist of four classes denoting chest cancer types i.e., adenocarcinoma, large cell and squamous cell carcinoma, and normal lung nodule. The images were first processed and converted into float values. Data augmentation techniques i.e., rotation range, width shift range, height shift range, shear range, zoom range, and horizontal and vertical flips are implied to increase its diversity to assist in the generalization of the models. The ResNet50 model is applied during the training of the dataset. The hyper-parameter configuration of the neural network architecture is optimally used for models’ enhanced performance. The hyper-parameters are listed in Table 4.

**Figure 4:**
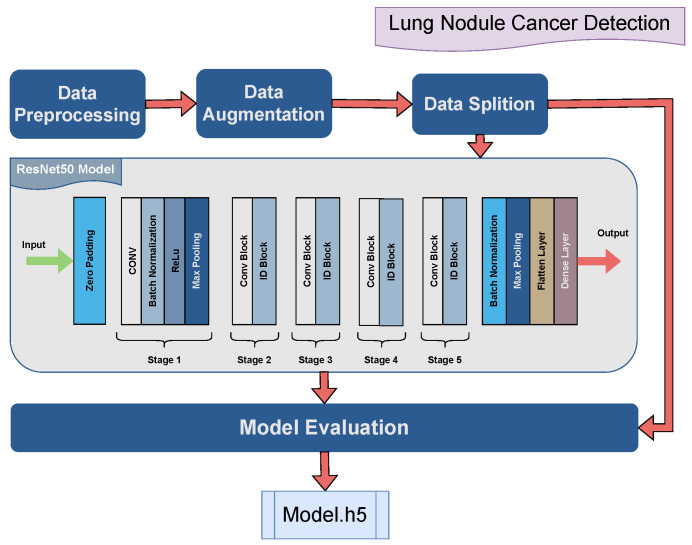
Graphical illustration of the proposed AI lung cancer detection model.

Figure 4 shows the proposed model which blends a pretrained ResNet50 CNN with customized fully connected layers for lung cancer classification. Initially, the ResNet50 model is set up while excluding its top layers and retaining ImageNet pretrained weights with its input shape. All layers within the ResNet50 model are untrainable in order to preserve their learned features during the subsequent training phase. Following this, a sequential model is implied to append additional layers atop the ResNet50 base. The layers include BatchNormalization for the normalization of activations, MaxPooling2D to downsample feature maps, and a Flatten layer to prepare the data for input into a dense layer to facilitate feature transformation and classification. These custom layers include varying numbers of neurons, ReLU functions, and dropout layers which are strategically inserted to mitigate overfitting by randomly dropping connections during training. The mathematical expression of the ReLU function is

(4) $$f\left(x\right)=\left\{\begin{array}{cc} x & \mathrm{if}\;x>0\\ 0 & \mathrm{otherwise}. \end{array}\right.$$

The dense layer uses the softmax to yield multi-class classification. The architecture effectively combines the robust feature extraction capabilities of ResNet50 with the flexibility of custom classification layers, thereby offering a powerful framework for medical image classification.

### 4.3. Brain Tumor Classification

The data are created by incorporating data images from figshare, SARTAJ, and Br35H datasets. The respective data used in the study contain 7023 MR images of human brain MR with four classes i.e., glioma, meningioma, pituitary, and no tumor. The data are initially processed with images being resized to (224, 224, 3), representing the shape of images being (224, 224) with three color channels (RGB). For data augmentation, data generators generate batches of image data to enhance model generalization with data shuffling during training and validation. The batch size is set to 16.

The ResNet50 model is applied during model training on the provided data. To enhance the model’s performance on the given MR image data, hyper-parameters (such as neurons, layers, and other model hyper-parameters) were tuned by trial-and-error technique. These hyper-parameters are shown in the following Table 5.

**Figure 5:**
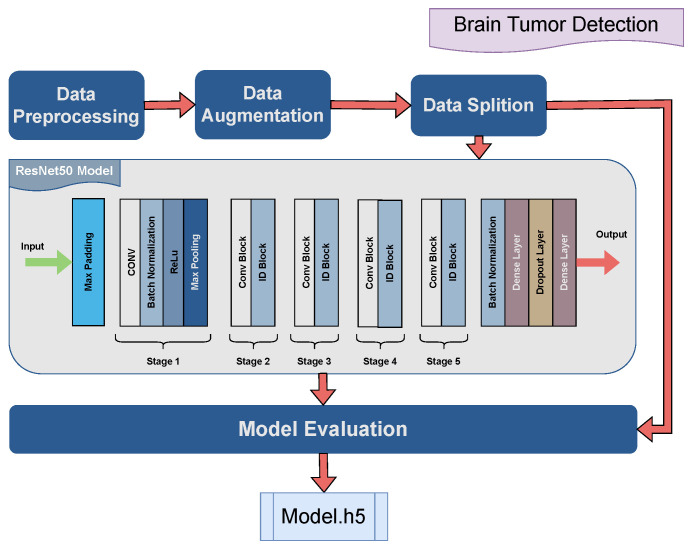
Graphical illustration of the proposed AI brain tumor detection model.

The proposed model is based on a convolutional neural network (CNN) architecture tailored for brain tumor classification by MR image as shown in Figure 5. It initializes a ResNet50 model pre-trained on ImageNet data, excluding its fully connected layers, and configures it to accept input images resized to 224 × 224 pixels with RGB channels. The custom head of the network is composed of layers to fine-tune the model for a particular classification task. Batch normalization is applied to normalize the activations, followed by a dense layer with ReLU activation and regularization terms, assisting in model generalization. A dropout layer is used with a probability range in 0.45 to 0.5. Finally, a dense output layer with softmax activation produces class probabilities corresponding to the number of classes in the dataset. The model uses an Adamax optimizer with a learning rate of 0.001 and the loss function used is categorical cross-entropy (CCE):

(5) $$\mathrm{CCE}\left(y,\hat{y}\right)=-\sum_{i=1}^{N}y_{i}\log\left({\hat{y}}_{i}\right)$$

where $y_{i}$ and $\hat{y_{i}}$ represent the probabilities of the ith class in the true and predicted distributions with *N* classes.

### 4.4. Skin Lesions Classification

The dataset used for training the proposed skin cancer detection model was primarily curated from ISIC-Archive data. The data contain a balanced set of microscopic images of benign and malignant skin moles. The accumulated data images were 1800 microscopic images focused on skin spots. The image data were processed into float32 for applying standard scaling to ensure uniformity and numerical stability. The shape of the images was resized to 224×224.

**Figure 6:**
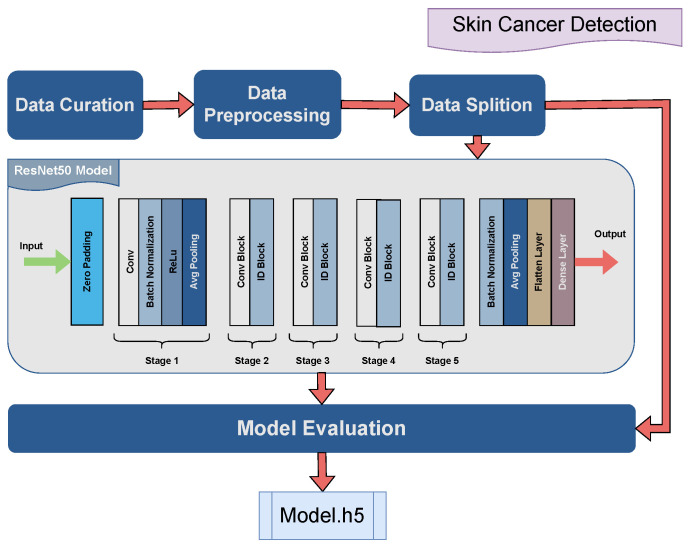
Graphical illustration of the proposed AI skin cancer detection model.

For model training, the ResNet50 model was applied to the given dataset. The hyper-parameters were fine-tuned to the model’s enhanced performance. The hyper-parameters for the proposed model are shown in Table 6. The proposed model architecture employed, shown in Figure 6, is centered around the ResNet50 CNN. The model’s setting consists of an input shape of (224, 224, 3) with 224 pixels for height and width and an RGB channel. The convolutional layer contains zero padding i.e., p=0 in Equation (2).

Global average pooling is utilized for spatial dimension reduction. The model is geared for binary classification with two output classes. Adam optimizer with a learning rate of 1 $\times \;10^{-5}$ is employed to optimize model weights. Binary cross-entropy (BCE) is used as a loss function and

(6) $$\mathrm{BCE}\left(y,\hat{y}\right)=-\left(y\log\left(\hat{y}\right)+\left(1-y\right)\log\left(1-\hat{y}\right)\right),$$

where *y* and $\hat{y}$ represent the true label and predicted label, respectively, which could be either 1 or 0.

The proposed architecture is tailored to binary classification of malignant and benign microscopic skin cancer moles, while emphasizing simplicity, efficiency, and adaptability to diverse data.

### 4.5. Renal Malignancy Classification

The data are utilized for the proposed architecture, which is curated by PACS with different classes of kidney tumor, cyst, normal, or stone findings [41]. Both the coronal and axial cuts were selected with both contrast and non-contrast studies from the abdomen and urogram. Each radiological finding was carefully selected from the Dicom study, one at a time, and a batch of Dicom images was created. Patient data and metadata from the Dicom images were extracted, which were then converted to jpg format. The dataset is comprised of 12,446 unique CT scan images which are distributed among four classes where the cyst class contains 3709, the normal class has 5077, the stone class has 1377, and the tumor class has 2283 images. The data were divided for training and testing with a 90:10 ratio. Then the pixel values of the images were normalized by dividing them by 255, resulting in values between [0, 1] to standardize the input data. Additionally, the datasets were also cached and prefetched using TensorFlow’s data pipeline functionality to optimize data loading performance and accelerate the training process. Caching allows the datasets to be stored in memory or disk after the first iteration, reducing data loading time for subsequent epochs. Prefetching further enhances efficiency by overlapping data preprocessing with model training, enabling a smoother and more seamless training process.

For training, a CNN model was implemented on the respective dataset to recognize the classes efficiently. Hyper-parameter tuning was performed rigorously to optimize the model performance. The proposed CNN model’s hyper-parameters are as shown in Table 7.

**Figure 7:**
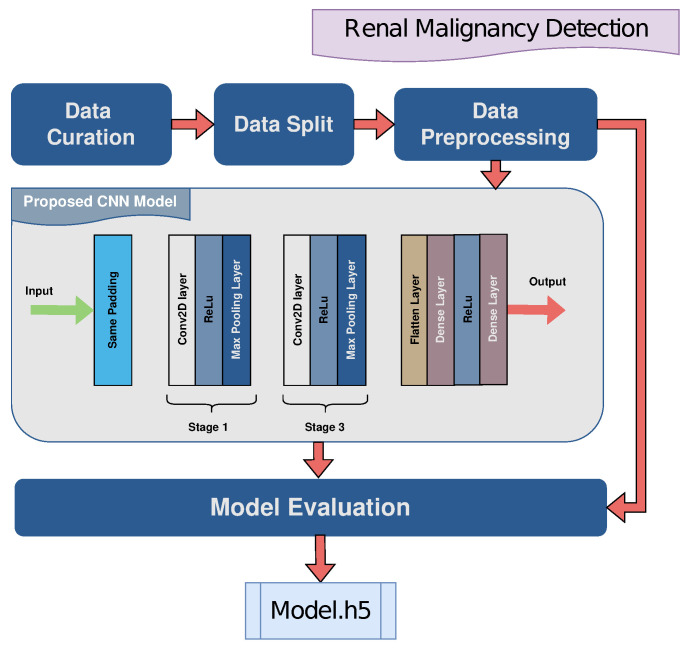
Graphical illustration of the proposed AI kidney malignancy detection model.

The model architecture depicted in Figure 7 is based on a CNN model designed for kidney malignancy classification. It consists of input with a convolutional layer with 32 kernels of size (3, 3) and ReLU. Input images are expected to have dimensions of 150×150 pixels and three color channels (RGB). Then, the max pooling layer with a pool size of (2, 2) extracts dominant features and reduces computational complexity. The mathematical expression for the output size of the convolutional layer with no padding as expressed in Equation (2).

Flattening converts high-dimensional feature maps to a single-dimension vector to connect it with the dense layers. The first one consists of 128 neurons while the final dense layer is comprised of four neurons, the required number of classes with softmax.

### 4.6. GPT-4 Precautions

GPT-4 is a cutting-edge AI model that has moved natural language processing in leaps and bounds. With its ability to learn from massive amounts of data and perform a wide range of language tasks, it can transform the way we communicate with machines. The model can be used to generate text according to the prompt that the user has inputted. The proposed GPT-4 architecture is used for generating medical assessments to help doctors make a more informed decision on the patient’s disease recognized by other AI models. GPT-4 is used in the study due to its mesmerizing results on medical assessments. In the years 2021 and 2022, three German medical licensing examinations were conducted among students and GPT-4, where GPT-4 achieved on average 85% accurate results where it stands percentile-wise as 92.8th, 99.5th, and 92.6th as compared to the medical students [42].

**Figure 8:**
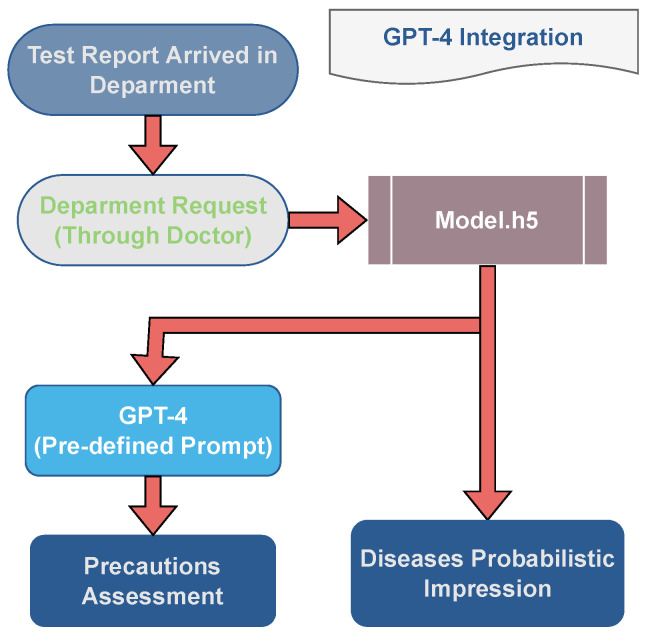
Graphical illustration of the proposed GPT-4 model system integration.

The prompt for generating optimized precautions for the respective disease was first fine-tuned and was applied depending on the departmental request for the disease assessment. Once the laboratory report is made accessible to the doctor, the doctor can then ask the GPT model to provide an AI-generated assessment. The system would process the doctor’s request by considering his department and applying an appropriate model for disease recognition and medical assessment as shown in Figure 8.

In Figure 8, the trained model first recognizes the malignancy through the inputted medical image. Simultaneously, GPT-4 then generates precautions for the medical professional while taking an optimized prompt and disease recognized by the AI model into consideration. This is an efficient way of generating disease-specific precautions which provide more valuable insights to the doctor rather than applying a generic prompt on each disease.

## 5. Models Performance Evaluation Settings

### 5.1. Accuracy

The accuracy illustrates the performance of a model over each training epoch or iteration. It plots the accuracy metric on the y-axis against a relevant independent variable, such as epochs, or iterations, while on the x-axis, parameter values are displayed. The graph’s trend reveals the accuracy changes concerning training epochs, which provides valuable insights into the model’s learning dynamics, convergence behavior, and optimal settings.

### 5.2. Confusion Matrix

The confusion matrix represents a statistical table for defining and illustrating the overall efficiency of an AI model [43] by evaluating a classification model’s performance by multiple statistical evaluating metrics as discussed in the following Section 5.3. A True Positive ($T_{P}$) represents correctly identified positive labels. On the contrary, True Negative ($T_{N}$) represents the condition when the model accurately identifies the negative labels. A False Positive ($F_{P}$) represents a specified condition where the model has incorrectly identified a positive label. Alternatively, a False Negative ($F_{N}$) defines incorrectly classified negative labels, as shown in Table 8.

### 5.3. Statistical Metrics

#### 5.3.1. Precision

This evaluation metric is used to measure the accuracy of positively predicted classes. High precision of a model usually indicates a more accurately predicted positive class. The mathematical formulation is as represented in Equation (7) [44]

(7) $$Precision=\frac{C_{c}}{C_{c}+C_{ic}}.$$

The range of values for precision is 0<Precision<1.

#### 5.3.2. Recall

Recall is used to indicate the ratio between correctly classified positive samples. A high value of recall usually represents the model’s adaptability for identifying positive class. The mathematical formulation is as shown in Equation (8) [44]

(8) $$Recall=\frac{T_{P}}{T_{P}+F_{N}}.$$

The output values of Recall can be 0<Recall<1.

#### 5.3.3. F1 Score

F1 score evaluates the model’s performance by harmonic mean considering precision and recall. Utilizing harmonic mean can penalize outlier values of either recall or precision, making it an efficient metric to assess a model’s overall performance. It is not symmetric among classes due to its dependence on positive or negative classes. Mathematically, it can be shown as [44]

(9) $$F1score=2\times \frac{Precision\times Recall}{Precision+Recall}.$$

The range of F1 score is 0<F1_score<1.

#### 5.3.4. Support

This statistical metric is used to quantify the number of actual labels of the classes in the respective dataset.

#### 5.3.5. Accuracy

Accuracy indicates the ratio of correctly classified and total samples in the data. However, it can be misconstrued, especially when addressing unequal class proportions. In this case, a model can achieve high accuracy by assigning all samples to the prevalent class, even though the model’s performance is substandard. Mathematically, [44]:

(10) $$Accuracy=\frac{T_{N}+T_{P}}{T_{N}+T_{P}+F_{N}+F_{P}},$$

where 0<Accuracy<1.

#### 5.3.6. Macro and Weighted Average

Macro-average is the average metric that averages out all classes. Alternatively, a weighted average assigns weights to the contributions of each class.

## 6. Results and Discussion

The evaluation of the proposed models is performed in three stages i.e., through (i) accuracy, (ii) confusion matrix, and (iii) classification report. The results from each model are compared with the other state-of-the-art models trained on the same dataset and the other conventional models.

**Figure 9:**
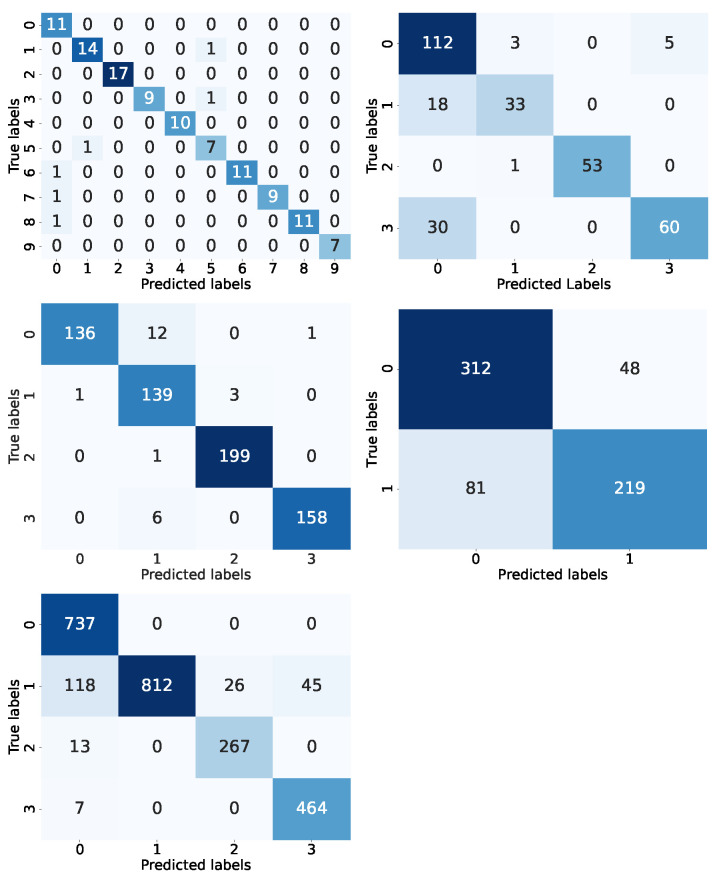
Graphical illustration of the confusion matrix for Bone Fracture recognition; Lung Tumor recognition; Brain Tumor detection; Skin Lesion identification; and Renal Malignancy recognition AI model.

**Figure 10:**
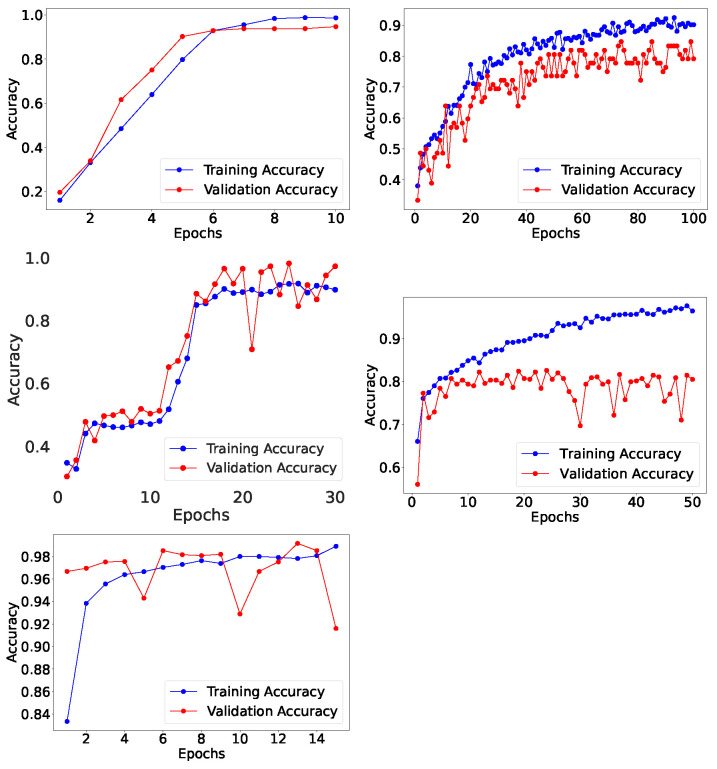
Graphical illustration of accuracy graph for Bone Fracture recognition; Lung Tumor recognition; Brain Tumor detection; Skin Lesion identification; and Renal Malignancy recognition AI model.

In Figure 9, the proposed CNN model is evaluated by confusion matrix by comparing the predicted and actual testing labels for the medical images. The model was evaluated by the statistical parameter as shown in the classification report in Table 9, where it shows more robust results than other models used for identifying bone fracture types such as MLP (Accuracy: 65%). With an accuracy of 95%, the proposed CNN model as shown in Figure 10 has outperformed several past models employed for bone fracture identification like BVLC Reference CaffeNet network/VGG CNN (Accuracy: 83%) [16], D-CNN (Accuracy: 89.2%) [18], CNN (Accuracy: 90.7%) [20], CNN (Accuracy: 79.3%) [26], and CNN (Accuracy: 90%) [35].

In Table 9, the proposed CNN model demonstrates high performance overall, with an accuracy of 95%. The macro average F1 score is 0.94, indicating a strong balance between precision and recall across the multiple classes. However, there is an imbalance in the recall value (0.00) for the “Greenstick fracture” class, which shows that the model struggles to correctly identify this type of fracture despite having perfect precision. Yet, “Spiral Fracture”, “Fracture Dislocation”, and “Hairline Fracture” classes have achieved perfect scores in all statistical metrics. the “Avulsion fracture” class has lower precision (0.79) but compensates with a recall of 1.00, showing the model’s ability to identify all positive cases, although it misclassifies some of the negative cases. Meanwhile, the “Comminuted fracture” class has a balanced performance of 0.93 for all statistical metrics.

Figure 9 shows the proposed ResNet50 CNN model evaluation performed by confusion matrix with a comparison of predicted and actual testing labels for the CT scans images of lung tumor. The perspective models employed for identifying lung cancer were also evaluated such as VGG-16 (Accuracy: 75%), VGG-19 (Accuracy: 78%), and FFNN (Accuracy: 52%), where the proposed ResNet50 CNN model performed superior with an accuracy of 90% as shown in Figure 10. The proposed model also outperformed several AI models in the literature like Ensemble learner of DCNN model (Accuracy: 84%) [19], TsDN (Sensitivity: 88.5%) [25], CNN (Accuracy: 86.67%) [29], and Faster R-CNN and transfer learning (Accuracy: 89.7%) [31], which were used for identifying lung cancer through a different kind of medical imaging, while the summary proposed model’s statistical evaluation is as shown in Table 10.

In Table 10, the proposed ResNet50 model shows strong overall performance with an accuracy of 90%. The model can identify “No Lung Tumor” class exceptionally well with a precision of 1.00 and a near-perfect recall value (0.98), showing that the model might rarely misclassifies non-tumor cases. The “Adenocarcinoma” class has a high recall value (0.95), which determines that the model detects most of the respective class cases, yet it has a low precision value (0.71), indicating some false positives. On the other hand, the “Large Cell Carcinoma” and “Squamous Cell Carcinoma” classes both have lower recall scores (0.67 each), which suggests that the model struggles to identify all instances of these tumors, but with precision values being relatively higher (i.e., 0.83 and 0.98, respectively).

In Figure 9, the proposed ResNet50 CNN model is evaluated by confusion matrix by comparing the actual and predicted labels from the testing MR images. The proposed model was also evaluated by the statistical parameter as shown in the classification report in Table 11 and compared with VGG-16 (Accuracy: 51%) for identifying brain tumors, where it shows robust results with an accuracy of 98% as illustrated in Figure 10. The proposed ResNet50 CNN model has outperformed other models from the literature as well i.e., SVM (Accuracy: 90.27%) [28], CNN (Accuracy: 84.19 %) [21], SVM (Accuracy: 86%) [22], Hybrid deep learning model (Accuracy: 96%) [32], and LSTM-CNN (Accuracy: 95.54%) [37] for identifying brain tumors.

In Table 11, the proposed ResNet50 model shows robust performance with an accuracy of 98% across 656 samples. High precision, recall, and F1 score values have been produced by the model for all four tumor classes, which indicates that the model is both highly accurate and effective at identifying the correct tumor class. The model has also achieved near-perfect results with a recall of 1.00 in the “no Tumor” class, which ensures that no false negatives are predicted. Meanwhile, the “glioma” and “pituitary” classes have F1 scores of 0.98 and show very balanced precision and recall values. However, the “meningioma” class has a slightly lower recall value (0.94), yet it still maintains a high F1 score of 0.95.

Figure 9 illustrates the proposed ResNet50 CNN model evaluation by confusion matrix by comparing the actual and predicted labels from the testing microscopic images for skin lesions. The proposed model was also evaluated by the statistical parameter as shown in Table 12, and compared with CNN (Accuracy: 70%) for identifying skin cancer, where it shows superior results with an accuracy of 97% as illustrated in Figure 10. The proposed ResNet50 CNN model has outperformed other past models as well employed for recognizing skin cancer images i.e., CNN (AUC: 91%) [17], CNN (Accuracy: 74%) [24], CNN (Accuracy: 86.65%) [36], XG-boost (Accuracy: 94%) [38], and Densenet169 CNN (Accuracy: 91.2%) [40].

In Table 12, the proposed ResNet50 model shows a balanced performance with an overall accuracy of 83%, as reflected in the micro, macro, and weighted averages. The model has slightly higher recall (0.88) than precision (0.83) value for detecting the “Benign” class, which means that the model might successfully identify most benign cases, though it occasionally misclassifies some instances as malignant. Meanwhile, for the “Malignant” class, the precision value (0.84) is higher than recall value (0.78), which indicates that the model is robust at predicting true malignant cases, yet it misses some instances.

Figure 9 presents the evaluation of the proposed CNN model using a confusion matrix that compares the actual and predicted labels from the testing CT scan images for renal malignancy. The proposed model was also assessed using statistical evaluators, as shown in Table 13, and compared with other models like VGG-16 (Accuracy: 96%) and MobileNet (Accuracy: 98%) in identifying renal malignancy. The proposed CNN model demonstrated superior performance with an accuracy of 99%, as illustrated in Figure 10. Furthermore, the proposed CNN model outperformed previous models used for recognizing renal malignancy, such as Inception-v3 (Accuracy: 88%) [27], ResNet (Accuracy: 60.4%) [33], Inception (Accuracy: 74.4%) [23], AlexNET (Accuracy: 91%) [30], and Inception-ResNet$V_{2}$ (AUC: 91.8%) [39].

In Table 13, the proposed CNN model exhibits an outstanding performance, with a near-perfect precision, recall, and F1 score value of 0.99 across all four classes with an overall accuracy of 99%. This evaluation of the model indicates that the model is highly effective at correctly predicting “Cyst”, “Normal”, “Stone”, and “Tumor” classes without any significant issues of false positives or false negatives. The consistent value across macro and weighted averages further confirms that the proposed model performs robustly regardless of the class distribution.

## 7. Conclusions

The proposed U-health system aims to digitalize healthcare by integrating patients, doctors, laboratories, pharmacies, and administrative personnel onto a single platform, enabling patient–doctor interactions, lab report generation, and online medication purchases. Central to this system is an AI-based healthcare component that processes medical images like X-rays, CT scans, MRI, and microscopic images to recognize diseases or malignancies, providing AI-generated precautions for doctors. It employs multiple DCNN models to detect various tumors and fractures in the body (e.g., brain, bones, lungs, kidney, skin) and uses a fine-tuned GPT-4 for generating detailed medical assessments. The AI models have shown robust performance through accuracy graphs, classification reports, and confusion matrices, with GPT-4 chosen for its superior capabilities in medical explanation, reasoning, memory, and accuracy.

The framework has the limitation of not having been able to incorporate AI models for other diseases related to medical fields such as pathology, cardiology, hematology, etc. for accurate assessment and diagnosis of a patient’s disease. The AI module could also consider other types of medical data such as generic data and patient historical and personal data to identify and generate personalized precautions more efficiently and thus help medical professionals to diagnose the disease more accurately.

For future consideration, the framework could include models with more accurate results in identifying the diseases or in generation precaution for patients. The proposed framework can also be improved by patient feedback and convenience for effective diagnosis and improving user experience with the proposed U-health platform. As the growth of a tumor puts a patient at great risk, an AI model could also be used to estimate the progression of the tumor within a patient’s body by analyzing his last CT scans or MR images. This could provide doctors and surgeons valuable insight into diagnosing or operating on tumors in the respective patient’s body.

## Figures and Tables

**Table 1:** Classification approaches employed on medical images to yield robust results.

Reference	Year	Damage Area	Imaging	Method	Dataset	Results
[16]	2017	wrist, hand, and ankle Fracture	X-ray and MRI	BVLC Reference CaffeNet network/VGG CNN	Danderyd’s Hospital	Accuracy = 0.83
[17]	2017	Skin lesions	Microscopic Images	CNN	129,450 clinical images	AUC = 91%
[18]	2018	vertebra fracture	CT scan	D-CNN	10,546 sagittal view 2D images from 1432 CT scans	Accuracy = 89.2%
[19]	2019	Lung Nodule	CT scans	Ensemble learner of DCNN model	LIDC-IDRI	Accuracy = 84.0%
[20]	2019	Diaphyseal Femur Fracture	X-ray	CNN	175 real-patients with abnormal and normal circumstances	Accuracy = 90.7%
[21]	2019	Brain Tumor	MRI	CNN	Figshare	Accuracy = 84.19%
[22]	2019	Brain Tumor	MRI	noise removal, edge detection, and contrast enhancement with SVM	Figshare	Accuracy = 86%
[23]	2019	Renal Cancer	four-phasic CECT	Inception	128 ccRCC & 51 RO	Accuracy = 74.4%
[24]	2019	Skin lesions	Microscopic Images	Image segmentation with CNN	ISIC 2017	Accuracy = 0.74
[25]	2020	Lung Nodule	CT scans	TsDN	LIDC-IDRI	Sensitivity = 88.5%
[26]	2020	Femoral neck Fracture	Radiographic images	CNN	calcaneus CT images	Accuracy = 0.793
[27]	2020	Renal Cancer	CT scans	Inception-v3	32 benign and 136 malignant patients data	Accuracy = 88%
[28]	2020	Brain Tumor	MRI	SVM	3 datasets	Accuracy = 90.27%
[29]	2021	Lung Nodule	CT scans	CNN	JSRT	Accuracy = 86.67%
[30]	2021	Renal Cancer	MRI: T2WI T1WI	AlexNET	203 ccRCC & 40 RO	Accuracy = 91%
[31]	2021	Lung Nodule	CT scans	Improved Faster R-CNN and transfer learning	Heilongjiang Provincial Hospital	Accuracy = 89.7%
[32]	2021	Brain Tumor	MRI	Hybrid deep learning model	ISLES2015 and BRATS2015	Accuracy = 96%
[33]	2021	Renal Cancer	MRI: T2WI	ResNet	77 ccRCC & 42 pRCC & 46 chRCC & 34 AML	Accuracy = 60.4%
[34]	2022	Lung Nodule	CT scans	Machine learning	LNDb	Accuracy = 94%
[35]	2022	Mandibular bone fracture	CT scans	CNN	686 patients with mandibular fractures	Accuracy = 90%
[36]	2022	Skin tumor	Microscopic Images	CNN	ISIC Data Archive	Accuracy = 86.65%
[37]	2023	Brain Tumor	MRI	LSTM-CNN	limited Dataset of 253 MR images from Kaggle	Accuracy = 95.54
[38]	2023	Skin Carcinoma	Microscopic Images	XG-boost	PH^2^ dataset	Accuracy = 94%
[39]	2023	Renal Cancer	CT scans	Inception-ResNet$V_{2}$	US-based hospital and patients of the MIDOR dataset	AUC = 0.918
[40]	2023	Skin lesions	Microscopic Images	Densenet169 CNN	HAM10000 and ISIC	Accuracy = 91.2%

**Table 2:** Technological aspects covered by the proposed tools and techniques.

System Aspect	Tools and Techniques
Data Retrieval	ORM
Model Deployment	Deploy AI models using .h5 file format
Web Server Communication	WSGI
AI-Driven Insights	Integrate AI assessment tool on dashboard
Database Management	SQL
Data Protection	GDPR Compliance
User Authentication	Django Auth
Backend Framework	Django
Security Features	Built-in security
Administrative Interface	Admin Interface

**Table 3:** Hyper-parameter configuration of the bone fracture detection model.

Hyper-Parameters	Optimized Values
Activation Function	SoftMax
Loss	Categorical Cross-entropy
Learning Rate	0.0005
Metric	accuracy
Shuffle	True
Batch Size	32
epochs	10
Verbose	0

**Table 4:** Hyper-parameter configuration of the lung cancer detection model.

Hyper-Parameters	Optimized Values
Activation Function	SoftMax
Loss	Categorical Cross-entropy
Optimizer	adam
Patience	5
Metric	accuracy
Weights	imagenet
epochs	100
Verbose	1

**Table 5:** Hyper-parameter configuration of the brain tumor detection model.

Hyper-Parameters	Optimized Values
Activation Function	SoftMax
Loss	Categorical Cross-entropy
Batch size	16
Learning rate	0.001
Optimizer	adamax
Weight	imagenet
Metric	accuracy
Pooling	Maximum
Epochs	30
Shuffle	False
Verbose	1

**Table 6:** Hyper-parameter configuration of the skin cancer detection model.

Hyper-Parameters	Optimized Values
Activation Function	SoftMax
Loss	binary cross-entropy
batch size	64
Learning rate	1 $\times \;10^{-5}$
Optimizer	adam
weight	none
Metric	accuracy
pooling	average
epochs	50
Verbose	2

**Table 7:** Hyper-parameter configuration of the renal malignancy detection model.

Hyper-Parameters	Optimized Values
Activation Function	SoftMax
Loss	Sparse Categorical Cross-entropy
batch size	351
Optimizer	adam
Metric	accuracy
epochs	15
Pooling	Max Pooling

**Table 8:** List of classification metrics.

Symbols	Metrics
$C_{c}$	Classified Correctly
$C_{ic}$	Classified Incorrectly
$F_{N}$	False Negative
$F_{P}$	False Positive
$T_{N}$	True Negative
$T_{P}$	True Positive

**Table 9:** Proposed CNN model’s classification report for bone fracture.

	Precision	Recall	F1 Score	Support
Spiral Fracture	1.00	1.00	1.00	7
Comminuted fracture	0.93	0.93	0.93	15
Avulsion fracture	0.79	1.00	0.88	11
Fracture Dislocation	1.00	1.00	1.00	17
Hairline Fracture	1.00	1.00	1.00	10
Greenstick fracture	1.00	0.00	0.95	10
Impacted fracture	0.88	0.88	0.82	8
Oblique fracture	1.00	0.90	0.95	10
Longitudinal fracture	1.00	0.92	0.96	12
Pathological fracture	1.00	0.92	0.96	12
accuracy			0.95	112
macro avg	0.95	0.94	0.94	112
weighted avg	0.95	0.95	0.95	112

**Table 10:** Proposed ResNet50 model’s classification report for lung cancer.

	Precision	Recall	F1 Score	Support
Adenocarcinoma	0.71	0.95	0.81	120
Large Cell Carcinoma	0.83	0.67	0.74	51
No Lung Tumor	1.00	0.98	0.99	54
Squamous Cell Carcinoma	0.98	0.67	0.79	90
accuracy			0.90	315
macro avg	0.88	0.82	0.83	315
weighted avg	0.86	0.83	0.83	315

**Table 11:** Proposed ResNet50 model’s classification report for brain tumor.

	Precision	Recall	F1 Score	Support
glioma	0.99	0.98	0.98	149
meningioma	0.97	0.94	0.95	143
no Tumor	0.99	1.00	0.99	200
pituitary	0.98	0.99	0.98	164
accuracy			0.98	656
macro avg	0.98	0.98	0.98	656
weighted avg	0.98	0.98	0.98	656

**Table 12:** Proposed ResNet50 model’s classification report for skin lesions.

	Precision	Recall	F1 Score	Support
Benign	0.83	0.88	0.85	360
Malignant	0.84	0.78	0.81	300
micro avg	0.83	0.83	0.83	660
macro avg	0.83	0.83	0.83	660
weighted avg	0.83	0.83	0.83	660

**Table 13:** Proposed CNN model’s classification report for renal malignancy.

	Precision	Recall	F1 Score	Support
Cyst	0.99	0.99	0.99	737
Normal	0.99	0.99	0.99	1001
Stone	0.99	0.99	0.99	280
Tumor	0.99	0.99	0.99	471
accuracy			0.99	2489
macro avg	0.99	0.99	0.99	2489
weighted avg	0.99	0.99	0.99	2489

## Data Availability

The integrated data of CT scan, MRI, X-ray, and microscopic imagery used to analyze this proposed framework are publicly available with DOI: 10.17632/5kbjrgsncf.3. Accessed on 11 July 2024 through the following link https://data.mendeley.com/datasets/5kbjrgsncf/3, accessed on 1 January 2020.

## Abbreviations

The following abbreviations are used in this manuscript:

ANN	Artificial Neural Networks
AI	Artificial Intelligence
API	Application Programming Interface
BRATS	Brain Tumor Segmentation
BVLC	Berkeley Vision and Learning Center
CNN	Convolutional Neural Networks
CSRF	Cross-Site Request Forgery
CT-Scan	Computed Tomography scan
e-health	Electronic Health
EHR	Electronic Health Records
FFNN	Feed Forward Neural Networks
GDPR	General Data Protection Regulation
GPT	Generative Pretrained Transformer
IDRI	Image Database Resource Initiative
ISLES	Ischemic Stroke Lesion Segmentation
ISIC	International Skin Imaging Collaboration
JSRT	Japanese Society of Radiological Technology
IT	Information Technology
LLM	Large Language Model
LIDC	Lung Image Database Consortium
MLP	MultiLayer Preceptron
MRI	Magnetic Resonance Imagining
ORM	Object-Relational Mapping
PACS	Picture Archiving and Communication System
R-CNN	Region-Based Convolutional Neural Network
REST	Representational State Transfer
SQL	Structured Query Language
TsDN	Two-step Deep Network
u-health	Ubiquitous Health
VGG	Visual Geometry Group
